# Systematic review of beliefs, behaviours and influencing factors associated with disclosure of a mental health problem in the workplace

**DOI:** 10.1186/1471-244X-12-11

**Published:** 2012-02-16

**Authors:** Elaine Brohan, Claire Henderson, Kay Wheat, Estelle Malcolm, Sarah Clement, Elizabeth A Barley, Mike Slade, Graham Thornicroft

**Affiliations:** 1 Health Service and Population Research Department, Institute of Psychiatry, King's College London, England SE5 8AF, UK; 2 Nottingham Law School, Nottingham Trent University, Belgrave Centre, Chauser Street, Nottingham NG1 5LP, UK

**Keywords:** Mental health problem, Disclosure, Employment, Employer, Systematic review, Meta-ethnography

## Abstract

**Background:**

Stigma and discrimination present an important barrier to finding and keeping work for individuals with a mental health problem. This paper reviews evidence on: 1) employment-related disclosure beliefs and behaviours of people with a mental health problem; 2) factors associated with the disclosure of a mental health problem in the employment setting; 3) whether employers are less likely to hire applicants who disclose a mental health problem; and 4) factors influencing employers' hiring beliefs and behaviours towards job applicants with a mental health problem.

**Methods:**

A systematic review was conducted for the period 1990-2010, using eight bibliographic databases. Meta-ethnography was used to provide a thematic understanding of the disclosure beliefs and behaviours of individuals with mental health problem.

**Results:**

The searches yielded 8,971 items which was systematically reduced to 48 included studies. Sixteen qualitative, one mixed methods and seven quantitative studies were located containing evidence on the disclosure beliefs and behaviours of people with a mental health problem, and the factors associated with these beliefs and behaviours. In the meta-ethnography four super-ordinate themes were generated: 1) expectations and experiences of discrimination; 2) other reasons for non-disclosure; 3) reasons for disclosure; and 4) disclosure dimensions. Two qualitative, one mixed methods and 22 quantitative studies provided data to address the remaining two questions on the employers perspective.

**Conclusions:**

By presenting evidence from the perspective of individuals on both sides of the employment interaction, this review provides integrated perspective on the impact of disclosure of a mental health problem on employment outcomes.

## Background

Disclosure or self-disclosure can be defined as the process of communicating information about oneself verbally to another person [1]. Mental health services users face difficulties in deciding whether to disclose a mental health problem in the employment context [2]. The recent (limited) restriction on pre-employment questionnaires by section 60 of the Equality Act 2010, has been a positive step in recognising that people with a mental health problem experience stigma and discrimination in finding work e.g.[3,4]. Furthermore, Sections 6, 15 and 20 of the Equality Act 2010 (re-enacting most of the Disability Discrimination Act 1995) prohibit unjustifiable less favourable treatment of those with a mental disability and requires an employer to make reasonable adjustments for them (in other jurisdictions often referred to as "accommodations"). Not everyone with a mental health problem will be considered as having a disability under the Act: it is limited to those who have an impairment that has a substantial and long-term adverse effect on their normal day-to-day activities (section 6(1) (b)) However, if there is a disability under the Act the person can only be brought within its ambit if the employer has, or could reasonably be expected to have, knowledge of the disability (section 15). Therefore, disclosure is a crucially important consideration for an employee in this situation. This recent change in legislation, along with an increased acknowledgement of the role of stigma as a barrier to work for individuals with a mental health problem, highlight the timeliness of this review.

The visibility of a stigmatised attribute is a key factor in how it influences the individual's social identity [5]. Sources of differentness which are not immediately apparent (e.g. sexuality) have been described as discreditable identities [6]. Mental health problems can be thought of as a concealable difference, although certain symptoms, or their behavioural manifestations, as well as medication side effects, can make mental health problems more visible and less concealable. The relative concealability of mental health problems means that these differences are often unobservable to potential employers and so job applicants and employees with a mental health problem have a level of choice regarding if and when to introduce this information.

A dichotomous view of disclosure (i.e. disclosure vs. non-disclosure) is inadequate to characterise the complexities involved in the process. Four dimensions of disclosure will be discussed in this review: 1) Voluntary or involuntary (is disclosure under the individuals control or is the illness visible in speech, behaviour or appearance); 2) Full or partial (which aspects of the illness to disclose); 3) Selectiveness (e.g. whether to disclose widely or to select individuals only); and 4) Timing of the point at which disclosure is made (e.g. at pre-employment stages or once employed).

This study aims to determine the following:

1. whether people with personal experience of a mental health problem believe that disclosing this will lead to unfavourable treatment in employment

2. factors associated with the disclosure of a mental health problem in the employment setting

3. whether employers view or treat job applicants or employees with a mental health problem less favourably than others

4. factors influencing employer's ratings of job applicants or employees with a mental health problem

## Methods

### Information sources

Eight bibliographic databases were searched: Psychinfo (via OVID); Medline (via OVID); Cumulative Index of Nursing and Allied Health Literature (CINAHL) (via EBSCHO); International Bibliography of the Social Sciences (IBBS) (via OVID); Sociological Abstracts (via CSA); Applied Social Science Index and Abstracts (ASSIA) (via CSA); Cochrane Library and Open System for Information on Grey Literature Europe (OpenSIGLE).

### Search strategy

The following terms were used: (Mental NEAR disorder* OR Mental NEAR ill* OR Psychiatric* NEAR disabil* OR Schizophrenia OR Bipolar OR Depression OR Anxiety) AND (Job OR Employ* OR Work OR Interview* OR Application OR Occupation* OR Personnel) AND (Disclos* OR Non-disclos* OR Conceal* OR Hire* OR Accomodat* OR Discriminat* OR Prejudice* OR Stigma* OR Satisf* OR Stress* OR Function*). All terms were searched using a multi purpose search (.mp) which retrieves papers which include the search term in the abstract, heading word, title, original title, MeSH subject heading and table of contents. An inclusive strategy was used to maximise the number of results obtained as relevant papers may not be routinely indexed. The search was modified for each search engine as necessary. Retrieved papers were limited to those reporting on human subjects, and to the review period 1990- August 2010. Further information on inclusion criteria are displayed in Table 1.

**Table 1:** Inclusion criteria for systematic review

Aspect of interest	Inclusion criteria for Q1 and Q2	Inclusion criteria for Q3 and Q4
1. Population	Person with a mental health problem such as would be considered disabled under the Disability Discrimination Act 2005.	Persons involved in hiring decisions or managing individuals with a mental health problem. Includes employer, HR professional, occupational health professional, students or others participating in these role

2. Context	The context included voluntary or supported/sheltered employment as well as competitive or paid employment.	Presents information on hiring/retaining individuals with a mental health problem in voluntary or supported/sheltered environment as well as competitive or paid employment.

3. Outcome	Provided evidence to address one of the below issues: 1. The disclosure beliefs of people with a mental health problem 2. The disclosure behaviours of people with a mental health problem 3. Factors related to disclosure	Provided evidence to address one of the below issues: 1. The disclosure beliefs of employers 2. The hiring/job short-listing behaviour of employers 3. Factors related to hiring/job short-listing behaviour of employers

4. Study type	Any type of study containing primary data (quantitative or qualitative). Review papers and other non-data based papers which fulfil inclusion criteria 1-3 were retained and reference list were searched for relevant data-based papers, however they were not included in the review	

5. Publication Type	Published journal papers or journal papers in press or unpublished dissertations or reports	

6. Language	All languages. Where an English version of the title was not available, translation was performed	

7. Time frame	Published 1990 to August 2010	

The reference lists of ten identified review papers were hand searched to identify additional papers [7-17]. The reference lists of all included papers were also searched

### Study selection

Citations were managed using Reference Manager Version 11 [18]. Titles and abstracts were screened for relevance by the primary reviewer (EB); where relevance was unclear, the full text was obtained. As a reliability check, the eligibility of 10% of all located papers was checked by a second rater (EM) and agreement measured using the weighted Kappa statistic. A record of all excluded papers and the reasons for exclusion was maintained.

### Data collection process

Data on study characteristics, findings and the methodological quality of the studies were extracted into Microsoft Word tables separately by EB and EM. The following information was extracted: 1) characteristics of study participants; 2) study design and aims; and 3) outcome measures or analysis procedures used. Any discrepancies in data extraction were discussed and resolved. At this stage, further papers were excluded if they did not help to address one of the four research questions.

### Methodological quality of studies

For quantitative studies, quality was assessed using seven criteria adapted from relevant quality assessment tools for surveys [19,20]. For qualitative studies seven quality criteria were adapted from relevant guidelines [21-23]. Studies were classified as high, moderate or low quality based on the degree to which they fulfilled these criteria. Both qualitative and quantitative studies were classified as high quality if at least six of the seven criteria were fulfilled. Studies were considered of moderate quality if four or five of the criteria were fulfilled, and were considered to be of low quality if less than four of the criteria were fulfilled. To be rated as high or moderate quality, the criteria not fulfilled needed to be judged unlikely to alter the conclusions of the study [24].

### Data synthesis

Data from qualitative studies which addressed Q1 (Disclosure beliefs) were further synthesised using meta-ethnography. Meta-ethnography is a method that *"involves induction and interpretation and in this respect it resembles the qualitative methods of the studies it aims to synthesise" *p.210 [25]. The aim of meta-ethnography is to synthesise qualitative research studies, using a qualitative method. The seven stages identified by Noblit and Hare were used [26]. This includes: 1) getting started; 2) deciding what is relevant to the initial interest; 3) reading the studies; 4) determining how the studies are related; 5) translating the studies into one another; 6) synthesising translations; and 7) expressing the synthesis. Data were managed using NVivo version 8 [27]. The results and discussion sections of each study were entered into NVivo, and verbatim examples from each study were coded using the original codes suggested by the authors. A master list of all codes used was produced and examined for connections. The examples from each study were then coded again using a reduced list of codes. After all transcripts had been coded in this way the master list was reviewed again and structuring of super-ordinate themes and sub-themes were considered. All studies were then coded using the revised list of super-ordinate themes and sub-themes. Each sub-theme was present in at least two studies, while a minimum of one of the sub-themes of each super-ordinate theme was endorsed by 50% or more of studies (8/17 studies). In this way, the super-ordinate themes and sub-themes were reflective of the included studies.

Data from the quantitative studies which addressed Q2 (disclosure factors), Q3 (employer attitudes and behaviours) and Q4 (employer factors) was synthesised by examining the tables of extracted information. Statistically significant relationships were examined and grouped, using descriptive headings suggested by the authors, where appropriate. Due to the heterogeneity of study methodologies included, meta-analysis was not conducted.

## Results

### Study selection

**Figure 1:**
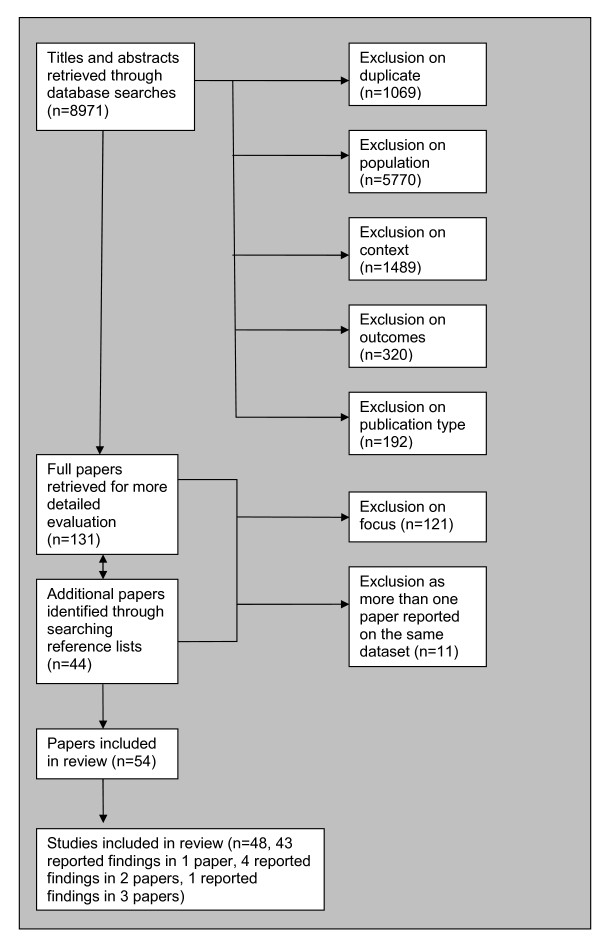
**Flow diagram for inclusion of studies**.

The search yielded 8,971 papers, as shown in Figure 1.

Fifty-four studies were identified and included in the review. In cases where more than one identified paper reported on the same source data, the papers were presented together as one study. Four sets of two papers and one set of three papers were identified which reported on different aspects of the same datasets. Each set was combined and reported together as one study, reducing the included number to 48. 23 papers concerned individuals with a mental health problem, 24 concerned employers, one paper concerned both. The first and second raters made the same judgment on the inclusion status of 873 (98%) of the 895 randomly chosen papers (weighted kappa = 0.69, *p *< 0.001), indicating an acceptable level of agreement.

Seventeen studies provided evidence on Q1 (disclosure beliefs). All were included in the meta-ethnography. Eight studies provided quantitative evidence on Q2 (disclosure factors). From these studies, five were excluded from this analysis for the following reasons: 1) One study was excluded from this section as the sample size (n = 20) was insufficient to accurately establish an association between variables [28]; 2) two studies were excluded as data on disclosure in individuals with a mental illness were only considered jointly with that from individuals with other chronic illness [29-31]; and 3) two studies were excluded as they provided only descriptive level data on disclosure [32,33].

Of the 25 reviewed studies, 10 were included in answering Q3 (employer attitudes and behaviours). Ten studies were excluded as they did not contain information to answer this question [34-43]. A further five studies were excluded as they contained descriptive but not inferential statistics [44-49]. Seventeen studies provided evidence to answer Q4 (employer factors). Eight studies were excluded in answering this questions as they did not contain relevant information [44-46,50-54].

### Evidence from individuals with a mental health problem (Q1 and Q2)

Additional file 1 provides details on 16 qualitative (refs 1-16), one mixed methods (ref 17) and seven quantitative (refs 18-24) studies. The included qualitative and mixed methods studies of people with a mental health problem had an average quality rating of 5.8/7 for the 17 studies; with all but one study in this group receiving a rating of at least moderate quality (4/7 or greater). The included quantitative and mixed methods studies of people with a mental health problem had an average quality rating of 5.3/7 for the 8 studies; with all studies in this group receiving a rating of at least moderate quality (4/7 or greater).

### Synthesis of results

#### Question 1. Employment-related disclosure beliefs and behaviours of people with a mental health problem

Using methods from meta-ethnography (as described in the data synthesis section), four super-ordinate themes were generated: 1) Expectations and experiences of discrimination; 2) Other reasons for non-disclosure; 3) Reasons for disclosure; and 4) Disclosure strategies. Each theme is discussed below with reference to quantitative literature on the topic, where relevant. Table 2 displays examples of quotes used to support each sub-theme and the frequency of endorsement of super-ordinate and sub-themes.

**Table 2:** Frequency of themes identified in the narrative synthesis of qualitative evidence

Themes identified	Example
**1. Expectation s and experiences of discrimination (n = 14 studies)**	

1.1. Wouldn't be hired (n = 8) Study numbers = 1, 2, 3, 4, 7, 9, 14, 17	"I don't think you'd get a foot in through the door that way. You wouldn't get taken on in the first place if you told them you had a big mental history" [55]

1.2. Unfair treatment in workplace (n = 4) Study numbers = 1, 2, 4, 5	"Because every time I go for a promotion interview, I'm always passed up. It's happened about 30 times by now... There's some kind of inherent risk involved. "We don't want to promote her-too much time off"..." [56]

1.3. Would lose credibility in eyes of others (n = 3) Study numbers = 2, 4, 11	"I can probably lose my credibility, but they wouldn't perhaps...that's something that they would just store away in their mind and not discuss. Well certainly not in front of me, maybe with others. And they would just put on a front of not disclosing any hypocrisy, I suppose..." [57]

1.4. Legislation does not provide protection (n = 3) Study numbers = 3, 4, 17	"[They] will find reasons apart from the disability for discriminating against potential employees if they know about the disability" "I would never, ever let on. I would make sure I had a good story for any time (such as gaps on a resume) that I might be asked about" [58]

1.5. Gossip (n = 4) Study numbers = 3, 12, 13	"I felt that people [at work] were...getting together and saying...'you mustn't talk to her'...and I don't think that's me being paranoid I think that's the way it was..." [59]

1.6. Rejection (n = 4) Study numbers = 6, 11, 14, 16	"I've had friends at work and... have told them because I thought they were friends...and it's like there's something scary about me, or, you know, I was diseased and it might be catching" [60]

**2. Other reasons for non-disclosure (n = 8 studies)**	

2.1. Passing (n = 4) Study numbers = 2, 6, 13, 15	"I don't go out and advertise myself as having a disability. I like being able to keep it hidden when I choose. I do run into problems, from time to time, when I then hear people talk about their true feelings about people with mental illness, and they don't know I've got one..." [56]

2.2. Illness as private (n = 4) Study numbers = 1, 2, 5, 17	"There's the whole thing about disclosure...I have to say that I was very private...that's like, " it's none of their business...You've got to balance privacy with accommodation"" [61]

2.3. Job with natural adjustments (n = 4) Study numbers = 1, 2, 4, 6	"Some jobs that I has really had the natural accommodations built into them. One was I was developing x-ray films and I was in a dark room and they 'd be sending these films to me and I'd be by myself most of the day, and I felt that was a great job because I didn't have that pressure..." [62]

2.4. Others don't want to know (n = 2) Study numbers = 4, 6	"...other people were off with back injuries, stubbed their big toe, whatever, but when I came back for being on, well you know, I'd say it was depression or whatever, no one wanted to talk about it and I felt further alienated" [60]

**3. Reasons for disclosure (n = 12 studies)**	

3.1. Role model for others (n = 2) Study numbers = 4, 12	"I like to think I've changed people's attitudes...I'd just explain to them what it were like. I said "this is what it were like for me' I said 'everyone 's not the same'...I said 'I'm not dangerous or anything or...' the only things they hear about are the ones on the news" [59]

**Themes identified**	**Example**

3.2. To gain adjustment (n = 3) Study numbers = 1, 4, 17	"Well I sort of feel like what's the point in disclosing, you have nothing to gain. (Laughs) I mean...I don't feel like I have anything to gain, especially during the job interview" [28]

3.3. Positive experience of disclosure (n = 5) Study numbers = 4, 6, 9, 13, 14	"It came up in one of our meetings I don't know why it came up but it came up and I mentioned it and she said 'Oh thank you for disclosing that', you know, 'I appreciate that..." [63]

3.4. To obtain emotional support (n = 3) Study numbers = 4,11,13	"A bit of understanding about how you might be feeling. A bit of... making allowances for the fact that today you don't feel good or today is a bad day for you or you have a good reason for not being happy today..." [57]

3.5. To be honest (n = 2) Study numbers = 2,11	"Oh just to be able to be honest and... have people's understanding of what it is to have any form of mental illness... it comes back to how you're brought up, community attitudes and values, basic morals, and that part of your personality. I think it's more personal than a group thing" [57]

3.6. To explain behaviour (n = 7) Study numbers = 3,6,9,11,13,14,16	"People used to make fun of it that I would come in so late in the morning. (I was) very well respected for my work, but, it began to be this problem of, '(respondent's name) is great, but why is he coming in at 10 o clock in the morning? Or 11 o clock?'"[60]

3.7. Stress of concealing (n = 4) Study numbers = 2,4,9,13	"I think to myself, you are ashamed of this, or you're worried about what people will think of you, because you have to invent this thing. Inventing it creates stress and it is using your mind to concoct all these things when it would be so much better to say, "you know what? I cannot do this because I have agoraphobia"..." [56]

**4. Disclosure dimensions (n = 9 studies)**	

4.1. Selective disclosure (n = 2) Study numbers = 1,3	"I think that what influences the decision is a pre-judgment on my part about the person, about their ability to hear me, so I'll like make a quick assessment if you will if there is anything about the person or the environment that is like could be oppressive or judgmental..." [62]

4.2. Partial disclosure (n = 5) Study numbers = 1,3,6,7,14	"Believe it or not, I've been on the job for seven months, and they do not know I have schizophrenia. They do not know I have a mental illness. They do know I have diabetes..." [64]

4.3. Inadvertent disclosure (n = 4) Study numbers = 2,3,4,14	"I'm a manic-depressive, so my manic state would go higher up and I'd be working constantly...it brought a lot of stress... when they fired me, they knew... they knew all along because I was hyper. They knew that they were going to let me go" [56]

4.4. Strategically timed disclosure (n = 3) Study numbers = 3,5,10	"...I just want to be known as me, you know...and until I feel more secure or confident that my peers wouldn't treat me as a different person, then I won't share that information in the workplace" [57]

Study numbers: 1 = [62], 2 = [58], 3 = [56], 4 = [65], 5 = [61], 6 = [60], 7 = [66], 8 = [67], 9 = [55], 10 = [68], 11 = [57], 12 = [59], 13 = [63], 14 = [69], 15 = [64], 16 = [54], 17 = [28]

##### 1. Expectations and experiences of discrimination

This super-ordinate theme was represented in 14 studies. There are six constituent sub-themes: 1) wouldn't be hired if disclosed; 2) unfair treatment in the workplace; 3) would lose credibility in eyes of others; 4) legislation does not provide protection; 5) gossip; and 6) rejection. The sub-theme 'wouldn't be hired if disclosed' represents the belief that a person would be treated unfavourably in finding work, if their mental health problem was known about'. Treated unfairly in the workplace' refers to beliefs or experiences of receiving less favourable treatment. This included a lack of benefits and reduced promotion prospects. Not wanting to be treated differently [70] and feeling stigmatised in the workplace [32] were represented in the quantitative literature. In the study by Lee and colleagues, 2007, 40.1% of participants with schizophrenia reported that their employers showed dissatisfaction with their taking sick leave [33]. 'Would lose credibility in eyes of others' describes beliefs or experiences of feeling devalued or undermined in the workplace as a result of others knowing about a mental health problem. With this loss of credibility comes a lowering of expectations about one's capacity to perform well in the job. The sub-theme 'legislation does not provide protection' refers to the belief that relevant anti-discrimination legislation is either personally irrelevant, or ultimately unsuccessful, in preventing discrimination. 'Gossip' represents beliefs or experiences that the person would become a target for gossip, if their illness was known. 'Rejection' describes beliefs or experiences of being rejected or ostracised in the workplace because of one's mental health problem. This was represented in four studies. Isolation from co-workers and breakdown of relationships were given as reasons for non-disclosure in the quantitative literature [70].

##### 2. Other reasons for non-disclosure

This super-ordinate theme was represented in eight of the included papers. There are four constituent sub-themes: 1) passing; 2) illness as private; 3) job with natural adjustments; and 4) others don't want to know. Passing refers to the processes of keeping a stigmatised identity successfully concealed. It originates from Goffman's work on the topic [6]. Passing can allow an individual to be treated the same as anyone else in the workplace. This sub-theme was represented in four studies. 'Illness as private' refers to the belief that information about mental illness is deeply personal and too intimate to share with individuals in a workplace setting. This was coded in four studies. Participants described having jobs which were highly compatible with their illness or jobs with natural adjustments. For some this involved working in a role in which having personal experience of a mental health problem was advantageous e.g. in mental health advocacy or support work. For others, the job was suitable as it offered the flexibility to work at home or was well suited to the skills of the individual. The final subtheme 'others don't want to know' represents the belief that people do not want to talk about mental illness and that telling others is a source of burden to the that person.

##### 3. Reasons for disclosure

This super-ordinate theme was represented in 12 studies. There are seven constituent sub-themes: 1) role model for others; 2) to gain adjustments; 3) positive experience of disclosure; 4) to obtain support; 5) to be honest; 6) to explain behaviour; and 7) concealing as stressful. The sub-theme 'role model for others' represents the beliefs that disclosure allows a person to educate others about mental illness and to be a role model for other individuals who are in a similar situation. This was particularly relevant for those who worked in positions within mental health services. Disclosure allows a person to request adjustments in the workplace. The sub-theme of 'to gain adjustments' also included those who indicated that they had no reason to disclose as they did not require any adjustments. Disclosure to gain an adjustment in the workplace (e.g. time off to attend medical appointments, change in work duties) was reported in the included quantitative studies [29,30,71]. The belief that it is best not to disclose if no adjustments are required was also represented in this literature. The subtheme 'positive experience of disclosure' included times when individuals reflected on positive experiences of disclosure e.g. if the recipient was understanding or made a reciprocal disclosure. The sub-theme 'to obtain emotional support' represents beliefs or experiences of obtaining support as a result of disclosure. This is presented as separate from formal adjustments in the workplace. The validity of this sub-theme is supported by the findings of Munir and colleagues (2005) who suggest that the belief that receiving support from a line manager in relation to a chronic illness is important was a significant independent predictor of disclosure in the workplace [29].

The sub-theme 'to be honest' represents beliefs including fear that lack of honesty could lead to dismissal as well as wanting to be proud of one's identity as a person with a mental health problem. For some participants disclosure was necessary to explain unusual behaviour or to stop colleagues from attributing unusual behaviour to attributes which were considered more stigmatising than mental illness e.g. taking illegal drugs. This was coded seven times. The difficulties that arise if a person does not explain their behaviour are also represented in this sub-theme. Lee and colleagues, 2006, provide quantitative evidence on this theme. In their Hong Kong study, 36% of the 320 participants reported being mistaken as lazy due to the side effects of psychotropic medication and 20% reported being mistaken as a drug addict due to medication side effects. The sub-theme 'stress of concealing' represents concealing a mental health problem in the workplace as a stressful experience. The experience of constructing a 'cover story' to explain unusual behaviour is described as a source of shame and an energy draining activity. This sub-theme was represented in four studies.

##### 4. Disclosure dimensions

This super-ordinate theme was represented in nine papers. There are four constituent sub-themes: 1) selective disclosure; 2) partial disclosure; 3) inadvertent disclosure; and 4) strategically timed disclosure. The sub-theme 'selective disclosure' represents using or wishing to use selective disclosure strategies. *'*partial disclosure' represents occasions where individuals disclosed highly selective information about their illness e.g. disclosing 'I have an illness' or 'I have a mental illness', without further information on the specific condition. As mentioned in the introduction, this can also encompass choosing to disclose a different illness e.g. depression when the diagnosis is schizophrenia, or disclosing only a physical illness when co-morbid physical and mental illnesses are present. 'inadvertent disclosure' represents accidental disclosure, brought about either by visible symptoms or carelessness such as blurting out something regarding illness. The quantitative literature discusses symptoms of illness or hospitalisation as sources of inadvertent disclosure [29,30,33,70,71]. The final sub-theme 'strategically timed disclosure' describes waiting until a point at which the person feels secure in their position, or with their colleagues, before disclosure.

#### Question 2. Factors associated with disclosure of a mental health problem in the employment setting

An increased likelihood of disclosure was significantly associated with the following factors:

1. **Gender**. Banks and colleagues found that women were significantly less likely to disclose than men, in the context of supported employment [71]

2. **Type of supported employment approach**. Participants on the Diversified Placement Approach reported more disclosure to supervisors and co-workers than those on Individual Placement and Support Programme [72]

3. **Emotional support**. Higher rates of disclosure to supervisors and co-workers were associated with higher perceived emotional support [72]

4. **Familiarity with legislation**. Increasing level of familiarity with the ADA was a significant predictor of disclosure [70]

5. **Having ever received state or disability benefits**. Having ever received state or disability benefits was a significant predictor of disclosure [70]

6. **Primary diagnosis**. Those who had a mood disorder were significantly less likely to disclose than those with schizophrenia [71]

7. **Severity and management of symptoms at work**. Those who displayed no symptoms at work were significantly less likely to have disclosed their illness [71]. Increasing duration of psychiatric medication use and decreasing level of capacity to regulate work in accordance with psychiatric condition were significant independent predictors of disclosure [70]

8. **Work setting**. Working in a mental health setting rather those in health/social services or technical/business/educational settings was a significant independent predictor of disclosure [70]

9. **Work related concerns**. Concern about losing one's job, feeling pressure to fit in and decreasing level of confidence about maintaining professional status, were all significant independent predictors of disclosure [70].

These data provide limited information on the factors associated with disclosure of a mental health problem in the employment setting. Two studies used unvalidated surveys, two were conducted in supported rather than mainstream employment contexts, and all were undertaken in one country (USA). The applicability of the findings in relation to the English context is not established. Overall, no strong quantitative evidence was found to support the relationship between disclosure and additional variables.

### Evidence from employers (Q3 and Q4)

Two qualitative, one mixed methods and 22 quantitative (n = 25) studies provided data for Q3 (Employers attitudes and behaviours) and Q4 (Employer factors). Information on these studies is presented in Additional file 2. The three included qualitative and mixed methods studies of employers received an average quality rating of 6/7, with all receiving a rating of at least moderate quality (4/7 or greater). The 23 included quantitative and mixed methods studies of employers received an average quality rating of 5.7/7, with all receiving a rating of at least moderate quality.

### Synthesis of evidence

#### Question 3. Are employers less likely to hire an applicant who discloses a mental health problem

In eight of the ten included papers, applicants with mental health problems were rated as less employable than either a candidate with a physical disability or a candidate with no disability in the following circumstances:

1. An applicant with a mental health problem (depression) was rated as significantly lower in suitability than an applicant with no known disability [73]

2. Applicants with depression were significantly less likely to be appointed compared with an applicant with a history of diabetes [74]

3. Applicants with back injury were rated more favourably in terms of expected job performance than those with a mental illness [75]

4. An applicant without a disability (single mother) received a significantly higher employability rating than the applicants with disabilities (acquired brain injury or schizophrenia). There was no significant difference between the two disability conditions in terms of employability [50]

5. A wheelchair using applicant was 7 times more likely to be hired than an applicant with a mental health problem (on medication for anxiety and depression) [76]. Previously depressed candidates were rated significantly less favourably in terms of recommendation for hiring than those with no disability [77]

6. There was a significant difference in positive responses (i.e. invitation to interview) for those who did not disclose a disability compared with those who disclosed depression [51]

7. There was a significant difference in employers attitudes to employing people with mental disabilities compared with physical disabilities [52]

In two papers no significant differences were found between applicants with a mental illness, physical illness or no illness:

1. There were no significant differences on hiring recommendations, competence, starting salary, activity and potency for applicants with paraplegia, epilepsy, depression or no disability [78]

2. There was no significant difference in hiring decisions by extent of disclosure (none, brief, detailed) or disability type (psychiatric, physical) [79]

Overall, the weight of evidence suggests that disclosure of a mental illness places job applicants at a disadvantage in securing employment compared to applicants with a physical disability or no disability. There are however, questions of validity in accepting this evidence as it is largely based on employer rating of vignettes as part of a survey study [41,50,52,74,76], or else participation in an experimental exercise [73,75,77,78]. There is limited information on how vignette responses or behaviour in a controlled experimental situation correspond to real life behaviour. In one included study experimentally manipulated job applications were sent in response to real job adverts [51]. The findings of this study are however limited due to insufficient reporting of statistics.

#### Question 4. Which factors influence employers' hiring beliefs and behaviours towards job applicants with a mental health problem

Factors are presented in four groupings: 1) job characteristics; 2) employer characteristics; 3) job applicant characteristics; and 4) organisational/interview related characteristics. Non-significant findings are not presented unless they proved contradictory evidence to another study reporting a significant finding.

##### 1. Job characteristics

*Job industry*. There were significant differences by industry classification in hiring concerns. Social Services employers had significantly lower concerns regarding symptoms than employers in the transportation, communication and utilities industry. Social services employers also had the highest proportion of having a company policy towards hiring people with disabilities [40].

*Level of responsibility*. The perceived likelihood of success in employment was influenced by the position for which the person was applying, with the difference for 'executive' applicants being greater than for 'clerical' or 'manual' positions [74].

##### 2. Employer characteristics

*Previous experience of employing someone with a mental illness*. Employers who had previous experience of hiring people with a mental health problem expressed lower concerns regarding the work performance and administrative performance of individuals with a mental health problem [40]. Employers with positive experience of employing individuals with mental illness were more willing to hire a person with mental illness [37]. Motivation to employ people with a psychiatric disability was significantly associated with prior experience of employing people with psychiatric disabilities [41].

*Employer knowledge of disability legislation*. Receiving formal information about the Americans with Disabilities Act (ADA), was significantly associated with compliance with the ADA in hiring decisions [47,48]. Hazer and Bedell alternatively found that participant ADA knowledge and attitude were not significantly related to applicant suitability ratings [73].

*Level of social contact with people with mental illness: *As the person with a mental health problem closest to the participant moved from 'self/close other' to 'distant other' the likelihood of having experience of employing someone with a mental health problem reduced. As it moved from 'self/close other' to 'nobody known/don't know' the likelihood was further reduced [42].

*Employer personality*. Those with high right-wing authoritarianism rated an applicant with schizophrenia as significantly less employable than the control candidate (no disability) [38].

*Traditionalism*. There was a significant relationship between level of traditionalism and willingness to employ a person with a mental illness [36]. Traditionalism was categorised as high, moderate (transitional) or low (modern).

##### 3. Job applicant characteristics

*Whether adjustments are required*. Applicants who requested an alteration of working hours were rated as significantly less suitable than those who did not [73]. Further information provided by Jackson and colleagues (2000) suggests that employers were concerned whether adjustments would be cheap and/or non-disruptive vs. expensive and disruptive. Knowledge of ADA predicted willingness to implement cheap/non disruptive adjustments while knowledge and attitude were both significantly associated with willingness to implement expensive/disruptive adjustments [39].

*Gender of applicant*. Male applicants with a disability were rated more favourably than female applicants with a disability on potency salary and activity in a supervisory job condition (office manager) while women were rated more favourably than men in a non-supervisory position (telephone salesperson) [78].

*Diagnosis type*. 54% of employers would never/occasionally employ someone who was currently depressed, 66% would occasionally/never employ someone with schizophrenia and 73% someone with alcoholism. There was a significant difference in these proportions [49].

##### 4. Organisational/interview related characteristics

*Geographical location*. Employers from Beijing mentioned 'not ever hiring people with a mental illness' more frequently than employers from Chicago or Hong Kong [35]. Location may be a proxy measure of other organisational characteristics such as the availability of employer resources

*Organisation size*. Large organisations were significantly more likely to employ current mentally ill people [49].

*Interview technique*. There was a significant positive association between using an interview technique with supplied benchmark answers for raters and positive hiring recommendation [77].

Significant relationships were reported between likelihood of hiring and a number of characteristics related to: 1) job characteristics; 2) employer characteristics; 3) job applicant characteristics and 4) organisational/interview related characteristics. However, it must be again cautioned that likelihood of hiring is a hypothetical variable based on experimental participants or completing a vignette based survey.

## Discussion

This review is timely because of recent legislative developments. Under the Equality Act 2010 there will be no disability discrimination if the employer does not know, or could not reasonably be expected to know about the disability. Not all people with mental health problems are disabled within the meaning of the Act, but establishing a disability discrimination case is the only significant route by which such an employee can challenge their employer's or potential employer's actions. This review synthesises what is currently known about this topic by considering literature from the perspective of both employers and individuals with a mental health problem. Available evidence was presented in relation to four issues: 1) the employment-related disclosure beliefs and behaviours of people with a mental health problem; 2) the factors associated with the disclosure of a mental health problem in the employment setting; 3) whether employers are less likely to hire applicants who disclose a mental health problem; and 4) the factors influencing employers' hiring beliefs and behaviours towards job applicants with a mental health problem.

The meta-ethnography presents a framework for considering disclosure beliefs and behaviours. It emphasises the complexity of disclosure decisions and the key role of stigma and discrimination. Limited information was located on the factors associated with disclosure of a mental health problem in the employment settings. A number of significant factors were presented, however the reliability of these findings was not conclusive, as discussed in the results section. There was evidence to suggest that disclosure of a mental health problem leads to hypothetical applicants receiving lower employment suitability ratings in vignette survey or experimental studies. However there was insufficient evidence to suggest that these findings can be extrapolated to make judgments regarding employer behaviour in real hiring situations. This is particularly the case when it is considered that 20% (5/25) of the included studies on employers used undergraduate students, as a proxy group who were asked to make decisions as if they were in the role of HR managers or employers. One of the studies which included both HR professionals and undergraduate students found that HR professionals assigned lower mean suitability ratings to candidates than the undergraduate students [73]. This suggests that studies using students may underestimate the degree to which employers' rate applicants who disclose a mental health problem as unsuitable for employment.

In considering the factors which influence employers' hiring beliefs and behaviours towards job applicants with a mental health problem significant relationships were reported between likelihood of hiring and a number of characteristics related to: 1) job characteristics; 2) employer characteristics; 3) job applicant characteristics; and 4) organisational/interview related characteristics. However, as discussed in the previous paragraph this is again limited by the use of students in the role of employers and the hypothetical nature of the experimental and vignette survey studies.

### Strengths and limitations

This review is strengthened by the use of quality indicators. The inter-rater reliability for 10% of identified papers indicated an acceptable level of agreement (weighted kappa = 0.69, *p *< 0.001). The use of qualitative and quantitative quality ratings further suggest that the majority of papers were of at least moderate quality. A second person also provided a rating on the quality of all included papers and the reliability of quality ratings was compared. This aspect of quality could be further strengthened by involving a second analyst in establishing the super-ordinate themes and sub-themes for the meta-ethnography, as well as to code all papers using the final framework. In the absence of the assessment of reliability of quality assessment and meta-ethnographic coding, the researcher discussed these aspects with a range of colleagues who had experience in conducting systematic reviews. Written and verbal feedback was obtained on the appropriateness of the quality indicators, the meta-ethnographic super-ordinate and sub-themes, as well as the final analysis. Quality was also established through transparency of methodology and grounding in examples.

As mentioned, data were included from all countries which limit the generalisability of the findings to the UK context. This decision was taken given the anticipated lack of data in this area which was established in an earlier scoping review.

### Research and policy implications

Overall, 25% (6/24) of the included studies considering the perspective of people with a mental health problem were conducted in the UK and 20% (5/25) of the employer studies were conducted in the UK. This suggests that overall, this is not an area of research which has received a large amount of research interest in the UK, particularly since the review considered the period back to 1990. This systematic review supports the need for further quantitative and qualitative work in the UK. Longitudinal studies would be particularly welcome as little is known about how employers' knowledge, attitudes and behaviours, change over time. Qualitative work with employers is also currently limited. The identification of diagnosis as a factor in employers' hiring decisions suggests that further work to understand the specific beliefs which are triggered by specific diagnoses. Attitude research with the general public suggests that different diagnostic labels are associated with different stereotypes and trigger different social reactions [80,81]. This could also be further explored qualitatively, from the perspective of individual with a mental health problem, by considering whether the thematic structure presented is equally applicable to individuals with diverse diagnoses.

The findings have implications for understanding disclosure of other chronic illnesses in the employment context particularly concealable illnesses including: HIV/Aids, cancer, epilepsy, diabetes and chronic pain. Recent work examining attitudes to cancer among employers and cancer survivors highlighted a discrepancy in beliefs between these two groups [82]. Further work to replicate the review in these areas would allow a comprehensive picture of disclosure of concealable health conditions.

Awareness among employers of what constitutes a mental health problem and of the prevalence of mental health problems had significantly improved between 2006 and 2009. However, this increase did not translate into an increased use of formal mental health policies in the workplace or an increase in employers' knowledge about the law regarding mental health in the work place [43]. This systematic review emphasises the need for employers' concerns to be discussed frankly with appropriate mechanisms to develop their confidence in their abilities to manage individuals with mental health problems [42]. Occupational health professional have a key role to play in this task [83]. By considering evidence on the workplace beliefs and behaviours of employers, occupational health advisors can dispel myths and address concerns regarding hiring, managing and working with individuals with a mental health problem. A recent review suggests that 'recovery-oriented' and 'see the person messages', may be particularly suitable for use in public health campaigns [84]. Similarly, targeting interventions around these messages may be useful for employers. This review further suggests a role for interventions that focus on workplace and non workplace social contact with individuals with a mental health problem, as well as a focus on clarifying current legislations.

## Conclusions

This review has considered the beliefs and behaviours of people with a mental health problem regarding disclosure in the employment context. It has also considered the beliefs and behaviours of employers or people with hiring responsibility. By presenting evidence from the perspective of individuals on both sides of the employment interaction, this review has provided an integrated perspective on the impact of disclosure of a mental health problem on employment outcomes. Furthermore, this information is of significance in assessing the effectiveness of the disability discrimination provisions of the Equality Act 2010 where protection is dependent upon whether the employee/potential employee has disclosed their mental disability to the employer.

## Competing interests

The authors declare that they have no competing interests.

## Authors' contributions

EB designed and completed this systematic review as part of her PhD research under the supervision of GT and MS. CH provided guidance on the psychiatric literature. KW provided guidance on the legal aspects of disclosure and contributed to the background section. SC and EAB provided guidance on systematic reviewing. EM contributed to second coding 10% of all studies and coding the quality of all included studies. All authors contributed to revising the manuscript. All authors read and approved the final manuscript.

## Pre-publication history

The pre-publication history for this paper can be accessed here:

http://www.biomedcentral.com/1471-244X/12/11/prepub

## Supplementary Material

Additional file 1 **Table S1**. Studies investigating disclosure beliefs, behaviours and associated factors. Click here for file

Additional file 2 **Table S2**. Studies assessing employers hiring beliefs, behaviours and associated factors. Click here for file
