# Supplementary material for: Systematic review of beliefs, behaviours and influencing factors associated with disclosure of a mental health problem in the workplace
Source: BMC Psychiatry. 2012 Feb 16;12:11. doi: 10.1186/1471-244X-12-11 (PMC3298486; doi:10.1186/1471-244X-12-11)
Supplement: Additional file 1 — Table S1. Studies investigating disclosure beliefs, behaviours and associated factors. [file 1471-244X-12-11-S1.DOC]

###### Supplementary Table 1: Studies investigating disclosure beliefs, behaviours and associated factors

| Reference | | Sample | Aims | Study design |
| --- | --- | --- | --- | --- |
| 1. (Dalgin & Gilbride, 2003) | | 6 men 5 women. All had been hospitalised for psychiatric reasons on 1+ occasion. 7 were currently employed. US | To examine how people with psychiatric disabilities describe disclosure experiences and to investigate the specific concerns of individuals regarding disclosure to employers | Qualitative. Focus group and individual interviews. Grounded theory approach |
| 2. (Allen & Carlson, 2003) | | 5 men 8 women. Diagnosis of rheumatoid arthritis, osteoarthritis, cancer, depression, HIV/AIDS, fracture of the wrist, traumatic head injury, intervertebral lumbar disc prolapse. Australia | To explore the experiences of workers with a range of disabilities who have made successful employment transitions and their views on concealment | Qualitative. Focus group, individual interviews and use of researcher’s log. Phenomenological approach |
| 3. (Goldberg et al., 2005) | | 16 men 16 women with psychiatric disabilities interviewed at time 1. 28 people interviewed at time 2, 18-24 months later. Equal numbers were employed and unemployed at the time of first interview. US | To examine how people with psychiatric disabilities navigate employment and disclosure | Longitudinal qualitative study. Semi-structured interviews. The first interview was face to face and second was by telephone. Data sorted into categories using NVivo. Each category was analysed and common themes and experiences identified |
| 4.(Owen, 2004) | | 10 men 10 women with a diagnosis of severe mental illness who were employed in a non-managerial, competitive job. US | To understand individual decision processes around disclosure of a psychiatric condition in the workplace | Qualitative. Individual interviews. Grounded theory approach |
| 5. (Auerbach & Richardson, 2005) | | 2 men 4 women. All had severe and persistent mental illnessand had worked in competitive employment for at least 18 months during the previous 3 years. US | To examine the work experiences of individuals with severe mental illness | Qualitative. Semi structured interviews. Grounded theory approach |
| 6. (Michalak et al., 2007) | | 11 men 22 women with a diagnosis of bipolar disorder. Canada | To provide a detailed examination of the ways in which bipolar disorder impacts on work functioning | Qualitative. Interviews. “Qualitative research methods” |
| 7. (Dinos et al., 2004) | | 46 people with a diagnosis of mental illness. UK | To examine mental illness related stigma and the consequences of stigma for the individual | Qualitative. Narrative interviews |
| 8. (Schulze & Angermeyer, 2003) | | 12 focus groups each with 8-12 participants. 3 with service users, 3 with relatives and 3 with mental health professionals. 25 services users, 31 relatives and 27 mental health professionals in total. 64% female, 36% male. All service users had a diagnosis of schizophrenia. Germany | To examine the experiences of stigma from the perspective of individuals with schizophrenia, their relatives and mental health professionals | Qualitative. Focus group study. Analysis involved inductive formation of categories from the texts |
| 9. (Marwaha & Johnson, 2005) | | 8 men 7 women with a diagnosis of schizophrenia or bipolar affective disorder. UK | To examine the advantages and disadvantages of work, the ways in which illness affects ability to work; experiences of looking for work and perceived barriers to finding and keeping it; opinions on service provision | Qualitative. Semi-structured interviews. Thematic Analysis approach |
| 10. (O'Day et al., 2006) | | 14 men 16 women with severe mental illness. All were working 18 hours+ per week and had been employed for 3 years+. US | To examine the strategies used by people in employment to maintain employment and build satisfying careers | Qualitative. In depth interviews. NVivo qualitative analysis software was used and themes identified |
| 11. (Joyce et al., 2009; Joyce, Hazelton & McMillan, 2007) | | 5 male 24 female nurses with a psychiatric diagnosis. Australia | To examine the workplace experiences of nurses who have a mental illness | Qualitative. In depth interviews. Discourse analysis and critical ethnography |
| 12. (Nithsdale et al., 2008) | | 4 male 4 female clients of a community mental health team. UK | To examine the experiences of employment among individuals with psychosis who have secured competitive employment | Qualitative. Interpretative Phenomenological Analysis |
| 13. (Boyce et al., 2008) | | 13 male 7 female clients of employment support agencies. UK | To examine mental health service users experiences of returning to competitive employment following supported employment | Qualitative. In depth interviews. Transcripts were analysed thematically following the steps advocated by Miles and Huberman (1984). |
| 14. (Bergmans et al., 2009) | | 6 women (2 co-facilitators and 4 experts by experience) who had taken part in a 20-week intervention for people with recurrent suicide attempts. Canada | To examine the experiences and barriers associated with return to paid employment following recurrent suicide attempts related to mental illness | Qualitative. Collaborative qualitative case study. Thematic narrative approach. analysis. |
| 15. (Killeen & O'Day, 2004) | | 16 men 16 women with a psychiatric disability. US | To examine the expectations and beliefs of individuals with psychiatric disabilities regarding employment | Qualitative. In-depth interviews |
| 16. (Hauck & Chard, 2009) | | 6 men, 3 were employees with personal experience of depression, 3 were in employer/manager positions. All worked for the same private sector forestry products company.  Canada | To examine how employers and managers perceive depression and its impact on work performance | Qualitative case study approach using in-depth interviews. A phenomenological approach was taken in the analysis. |
| 17. (Gioia & Brekke, 2003) | | 10 male, 10 female young adults with recent-onset schizophrenia. All had worked competitively before diagnosis and returned to work afterwards. US | To examine knowledge and use of the Americans with Disabilities Act and work-related experiences adults | Mixed methods. Semi-structured interviews. |
| 18. (Ellison et al., 2003) | 495 participants. All had a serious mental illness and were employed in a professional or managerial position for at least 6 months in the past 5 years. Those who worked in mental health self-help and advocacy settings were excluded. Self-employed people were also excluded leaving a subsample of n=350 (67% women). US | | To examine patterns and correlates of workplace disclosure among professionals and managers with psychiatric conditions | Survey created for this study. Purposive sampling. Response rate 66.5% |
| 19. (Munir et al., 2005; Munir et al., 2006) (Munir, Jones, Leka & Griffiths, 2005) | 461 women 273 men with a chronic illness. All were employees of a UK university. Illness was grouped as asthma, arthritis, IBS, migraine, diabetes, depression and anxiety, musculoskeletal pain and heart disease. 17% of women and 20% of men were in the depression & anxiety category. UK | | To examine predictors of disclosure of a chronic illness at work (2005); to examine gender differences in self-management and disclosure of a chronic illness at work | Postal survey via university via internal mail. Response rate 44% |
| 20. (Banks et al., 2007) | Survey sent to 243 people (response rate 67%). Survey completed on behalf of 162 people participating in 10 supported employment programmes (62% male). This was completed as a follow up stage of a study on workplace supports and integration outcomes for supported employees. US | | To examine disclosure of a psychiatric disability in the context of supported employment | Survey. Measure development.  A five items disclosure supplement was developed |
| 21. (Ellison et al., 2008) | 347 individuals (63% women) with severe mental illness who had succeeded in obtaining and retaining mid to upper level professional or managerial positions. US | | To examine the capacity of individuals with severe mental illness to function in professional and managerial jobs and examine the correlates of employment outcomes in this sample | Postal survey created for this study. Ways of coping questionnaire also administered. Response rate 58% |
| 22. (Munir et al., 2007) | 1029 employees (48% male) managing either musculoskeletal pain  (n=324), arthritis and rheumatism (n=192), asthma (n=174), depression and anxiety  (n=152), heart disease (n=96) or diabetes (n=91). Participants were workers from four organisations across three sectors: local government, transport and manufacturing. UK | | To examine psychosocial factors associated with psychological and health-related distress among employees with a chronic illness | Cross-sectional questionnaire data. Items were measured on a five point Likert scale (not at all to full disclosure), and had an internal consistency α =.89 |
| 23. (Lee et al., 2006) | 320 outpatients (68% men, 32% women) with schizophrenia who resided in 28 halfway houses and a comparison group of 160 outpatients with diabetes mellitus at a public clinic. Hong Kong | | To examine the personal experience and structural context of treatment-related stigma among Chinese patients with schizophrenia in Hong Kong | Survey development. 28-item questionnaire developed for the study, based on focus groups. Internal consistency α =.79 |
| 24. (Rollins et al., 2002) | 104 employed clients on 2 types of supported employment programme. 45 from Diversified Placement Approach and 59 from Individual Placement and Support. All participants had a diagnosis of severe mental illness. US | | To examine the association between supported employment model and characteristics of workplace social relationships with supervisors and co-workers | Interview and survey based. The workplace network grid was used to assess perceived social relationships on the job. Work placement scale was used to classify level of employment |
