# Supplementary material for: Systematic review of beliefs, behaviours and influencing factors associated with disclosure of a mental health problem in the workplace
Source: BMC Psychiatry. 2012 Feb 16;12:11. doi: 10.1186/1471-244X-12-11 (PMC3298486; doi:10.1186/1471-244X-12-11)
Supplement: Additional file 2 — Table S2. Studies assessing employers hiring beliefs, behaviours and associated factors. [file 1471-244X-12-11-S2.DOC]

###### Supplementary Table 2: Studies assessing employers hiring beliefs, behaviours and associated factors

| **Reference** | **Sample** | **Aims** | **Study design** |
| --- | --- | --- | --- |
| 1. (Tse, 2004) | 72 business owners or human resource managers. New Zealand | To investigate employers perspectives on:   1. Employing people with mental health issues 2. Accommodating employees and job applicants needs in the workplace | Qualitative. Semi structured interviews |
| 2. (Hauck & Chard, 2009) | Six men, 3 were employees with personal experience of depression, 3 were in employer/manager positions. All worked for the company.  Canada | To examine how employers and managers perceive depression and its impact on work performance | Qualitative case study approach using in-depth interviews. A phenomenological approach was taken in the analysis. Credibility and dependability of research were assessed |
| 3. (Tsang et al., 2007). (Corrigan et al., 2008) | 40 employers in the US, 30 from Hong Kong and 30 from Beijing from small size firms (3-100 employees).  Employers were selected from 6 industries: high technology, low technology, education, healthcare, business, and manufacturing.  US, Hong Kong and China | Cross-cultural comparison of employers concerns about hiring people with psychotic disorder.  Specific focus on:   - - 1. The qualities they consider most important in hiring an employee     2. Specific concerns about hiring a person with a psychotic disorder for the position. | Mixed methods. Semi-structured interviews. Content analysis.  Companies were identified through the yellow pages and were cold-called to introduce the study and its aims |
| 4. (Hazer & Bedell, 2000) | 112 undergraduate psychology or public affairs students and 32 HR professionals. US | To investigate the effect of seeking reasonable accommodations and disability type on employment suitability ratings. | Experimental. 2x3.Candidate help seeking (in the form of a reasonable accommodation) vs. non help seeking was crossed with disability type (none, physical (kidney dysfunction), psychiatric (depression)) |
| 5. (Glozier, 1998) | Sampled 200 Human Resource Officers in public limited companies sampled. 84 questionnaires were received (42% response rate). UK | To examine whether human resource officers hold stigmatising attitudes towards employing people with a diagnosis of depression.  To examine the beliefs on which these attitudes were based | Postal survey with vignette. Vignettes were identical except for the manipulation of an illness variable (diabetes vs. depression). Survey questions measured applicant characteristics, employment prospects, expectations of job performance, predicted sickness absence and credibility (the degree to which sick notes for this condition are believed), all rated on various 4-point Likert scale |
| 6. (Scheid, 1999). (Scheid, 2005) | 117 employers from businesses in a major southern metropolitan area of the US | To investigate the knowledge employers have about the ADA, compliance with the ADA, employment practices, the role played by stigma in employing individuals with mental illness | Telephone survey. Items asked about company’s knowledge and awareness of the ADA, employment policies, experiences, work environment and community attitudes towards the mentally ill |
| 7. (Hand & Tryssenaar, 2006) | 58 small business employers (1-29). Canada | To investigate the beliefs of small business employers regarding the employment of individuals with mental illness | Surveys and individual interviews. A modified version of the attitudes towards the employability of people with severe mental handicaps scales was used. |
| 8. (Gouvier et al., 2003) | 295 undergraduates with business-related majors. US | To assess the impact of access discrimination on 4 categories of disabling condition: closed head injury, chronic mental illness, developmental disability and back injury.  To assess the relationship between job complexity and hiring decisions in disability.  To assess the interaction between amount of public contact and disability type | Experimental. 4x2x2 mixed methods design. The position was either a janitor or a phone operator (low vs. high complexity). Work was either during the day or at night (high vs. low visibility) |
| 9. (Fenton et al., 2003) | Questionnaire was sent to 174 companies; there was a 32% response rate. 23 surveys were completed by small companies and 32 surveys were completed by larger companies. UK | To examine employers’ attitudes to interviewing and hiring participants with a history of mental illness. In particular whether a previous admission under the Mental Health Act, 1983, has an impact. | Postal questionnaire |
| 10. (Bell & Klein, 2001) | 88 Full-time workers and 98 undergraduates. US | To examine the impact of disability (paraplegia, epilepsy, depress, no disability), gender and job level (supervisory vs. non supervisory) on job applicant ratings | Experimental 4x2x2. Job: telephone salesperson vs. office manager. Participants were rated on hiring recommendations, competence, starting salary, activity and potency |
| 11. (Fodor, 2006) | 32 male and 32 female university students took part in each of the control and experimental groups. Students completed the right wing authoritarianism scale with those in the top and bottom 27 percentile for authoritarianism included. US | To examine the relationship between right-wing authoritarianism and assessment of a person with paranoid schizophrenia (described as medically controlled) for a position as a manager of a research group within a biotech company | Experimental. Right wing authoritarianism scale (Duckitt, 2001). The scale is formed of 2 dimensions: social conformity and tough mindedness. Participants completed viewed a videotaped interview of a job applicant. 2x2 design |
| 12. (Herzig & Thole, 1998) | 46 employers. Malawi | To assess the attitudes of employers to hiring individuals with mental illness | Survey, hand delivered.13 items survey developed as part of this study. The questionnaire gathers information on the size of the workforce, staff absentee rates, attitudes and policies of employers towards employing people with a history of psychiatric problems |
| 13. (Manning & White, 1995) | Sampled [ersonnel directors at 200 companies. The 200 companies were a random sample of those registered with the stock exchange. 120 were completed for a 60% response rate. UK | To assess attitudes towards employing people who had been or were mentally ill | Survey. Instrument design. 17-item questionnaire was designed to assess attitudes towards employing people who had been or were mentally ill |
| 14. (Bricout & Bentley, 2000) | Survey posted to 1000 employers. 248 surveys were returned completed. Response rate of 25%. US | To investigate the impact of disability status on the perceived employability of job applicants | Postal survey with vignette sent to members of a national association of human resource managers. The applicant was experimentally manipulated on three conditions: 1) a single mother; 2) an applicant with an acquired brain injury (severe physical disability); and 3) an employee with schizophrenia (severe psychiatric disability). No mention was made of childcare or accommodation related needs |
| 15. (Koser et al., 1999) | 200 human resource personnel with the financial services sector. 73 (37% response rate) completed the survey. US | To compare employee selection among applicants with physical and mental disabilities.  To examine the impact of job type, boundary spanning vs. internal was also considered. A boundary spanning job is one which requires contact with outsiders e.g. customers, suppliers | Survey. Vignette. Applications were sent for the position of receptionist and credit analyst. One applicant was wheelchair bound while the other disclosed that she is currently taking medication for anxiety and depression. Participants received both applicants and were asked their preference |
| 16. (Jackson et al., 2000) | A sample of 200 organisations was selected from a database of UK companies (67 small, 67 medium, 66 large companies).77 responded (response rate 39%). UK | To investigate personnel directors and managers’ willingness to change their current selection procedures in relation to their attitude towards the DDA, 1995, and attitude towards disabled people | Postal survey |
| 17. (Reilly et al., 2006) | Experiment 1: 166 undergraduates.  Experiment 2: 133 undergraduates.  US | To examine the way in which benchmarks (i.e. a standardised way of rating performance) affect perceptions of prior disability in an employment interview. The emphasis throughout was on previous psychiatric disabilities rather than current. | Experimental |
| 18. (Diksa & Rogers, 1996) | 373 employers. All employers had 20 or more employees. US | Development and initial testing of the Employers Attitude Questionnaire. | Telephone administered survey. Scale development. The scale measure assesses employer concerns about hiring persons with psychiatric disability and was developed after literature review, piloted with 10 employers |
| 19. (Dalgin & Bellini, 2008) | 60 employers. US | To provide insight into the effects that disclosing an invisible disability may have on the employment interview | Vignette study. Employers were shown a video of a candidate applying for an accountant position.  3 x 2 factorial design. The first variable was disability status: 1) no disability vs. 2) insulin dependent diabetes (invisible physical disability) vs. 3) bipolar disorder (invisible psychiatric disability) |
| 20. (Haj-Yahia, 1999) | Stratified random sample of 262 male employers. These individuals were selected from 1701 potential employers identified in the telephone directory. Israel | To examine the attitudes and willingness to employ people with a mental illness, among Muslim employers in Arab society | Survey. Measures of traditionalism, perceptions regarding inadequate relationship with God, perceptions of mental illness as a divine punishment, divine will, attitudes towards the mentally ill, rejection of the mentally ill and willingness to employ a mentally ill person, were included. Arabic language versions of questionnaires were developed and adapted as part of the study |
| 21. (Pearson et al., 2003) | 409 x 4 letters were sent out to prospective employers in response to advertised clerical positions. Hong Kong | To examine whether applicants with a disability (walking with the aid of crutches, reactive depression, hearing impairment) would receive fewer job offers than applicants without a disability | Experimental. 4x1 factorial design. The first variable was disability status: 1) no disability vs. 2) walking with the aid of crutches vs. 3) recovery from period of reactive depression vs. 4) hearing impairment. Other factors were held constant. Researchers applied for real jobs. 409 x 4 letters were sent out to prospective employers in response to all suitable clerical positions advertised in 2 newspapers over 3 months |
| 22. (Ozawa & Yaeda, 2007) | 358 employers. 43 participated in an additional component to assess the test-retest reliability of the survey. Japan | To examine employer attitudes towards employing people with psychiatric disabilities in Japan. To develop a psychometrically valid measure for assessing employer attitudes | Postal survey. Survey development. Response rate 22.4%.Participants completed the developed Attitudes towards Employment of Psychiatric Disability Scale. Questions on employer and organisational characteristics were also included |
| 23. (Brohan et al., 2010; Little et al., 2010) | 502 employers. 301 small companies (defined as those with 1-50 employees) and 201medium to large companies (defined as those with 51 or more employees). UK | To investigated whether employers who have experience of employing people with mental health problems differ significantly from those without such experience in terms of their knowledge, attitudes and behaviours regarding mental health in the work place(Brohan et al., 2010). Results were compared with a similar survey conducted in 2006 (Little et al., 2010) | Telephone survey. Questions were based on previously used questions (Diksa & Rogers, 1996; Fenton et al., 2003; Hand & Tryssenaar, 2006; Hazer & Bedell, 2000; Manning & White, 1995) |
| 24. (Zissi et al., 2007) | 102 employers with small retail trade or service businesses. Greece | To examine Greek employers attitudes towards employing people with disabilities | Face to face interviews. Survey instrument developed for the study |
| 25. (Gilbride et al., 2000) | 200 Midwestern and Southeastern employers  US | To examine employers’ attitudes towards hiring people with disabilities and towards vocational rehabilitation | Telephone survey. The Employer Hiring Practices and Perceptions Survey was developed and piloted for the survey |
